# Smartphone-assisted upconversion nanoparticle assay for rapid multiplex detection of H5, H7, and H10 avian influenza viruses

**DOI:** 10.1080/22221751.2025.2602315

**Published:** 2025-12-09

**Authors:** Jun Zhang, Han Wu, Ping Wang, Jiamin Fu, Xinyao Zheng, Fang Wan, Man Hu, Fumin Liu, Linfang Cheng, Hangping Yao, Nanping Wu, Junli Zhu, Haibo Wu

**Affiliations:** a State Key Laboratory for Diagnosis and Treatment of Infectious Diseases, National Clinical Research Center for Infectious Diseases, the First Affiliated Hospital, School of Medicine, Zhejiang University, Hangzhou, People’s Republic of China; b College of Food Science and Biotechnology, Food Safety Key Laboratory of Zhejiang Province, Zhejiang Gongshang University, Hangzhou, People’s Republic of China; c School of Statistics and Mathematics, Zhejiang Gongshang University, Hangzhou, People’s Republic of China

**Keywords:** Avian influenza virus, upconversion nanoparticles, multiplex immunochromatographic assay, ultrasensitive detection, smartphone

## Abstract

Avian influenza viruses (AIVs) of the H5, H7, and H10 subtypes pose substantial threats to global public health owing to their high pathogenicity, cross-species transmissibility, and potential to spark epidemics. Rapid and accurate detection is essential for outbreak control and zoonotic risk mitigation. Here, we report the development of a multiplex lateral flow immunoassay (LFA) based on core-shell upconversion nanoparticles (UCNPs) conjugated with subtype-specific monoclonal antibodies targeting the haemagglutinin proteins of H5, H7, and H10 AIVs. The assay achieved limits of detection of 0.0313, 0.0156, and 0.0625 ng/mL for recombinant HA proteins and 2^−4^, 2^−4^, and 2^−3^ haemagglutination units for viral titres of H5, H7, and H10, respectively. No cross-reactivity was observed with other AIV subtypes or respiratory pathogens, and intra- and inter-assay variation remained below 6%, demonstrating high specificity and reproducibility. Validation with 135 avian and 125 human clinical samples showed complete concordance with real-time RT-PCR results. Integration with a smartphone-based analytical platform enabled rapid readout, automated quantification, and cloud-based data sharing, providing results within 10 min. This intelligent UCNPs-LFA system combines ultra sensitivity, multiplexing, and field-deployable usability, representing a significant advance over conventional methods. By enabling timely and reliable detection of H5, H7, and H10 AIVs in both animal and human samples, this platform offers a practical tool for early warning, surveillance, and control of emerging zoonotic influenza, thereby contributing to global preparedness against avian influenza outbreaks.

## Introduction

Avian influenza viruses (AIVs), particularly highly pathogenic strains, remain a major threat to global public health due to their capacity for cross-species transmission and severe clinical outcomes in humans [1]. To date, 18 haemagglutinin (HA) and 11 neuraminidase (NA) subtypes have been identified, among which the H5, H7, and H10 subtypes have received increasing attention because of their zoonotic potential [2]. Since the first confirmed human case of H5N1 in 2006, multiple outbreaks have occurred in China and elsewhere, causing high mortality and sporadic human fatalities [3,4]. Similarly, the H7N9 subtype, first detected in 2013, has triggered at least five epidemics in China, with numerous human infections and a case fatality rate of approximately 39% [5]. More recently, the first human infection with H10N3 was reported in China in 2021, involving a novel reassortant virus with dual receptor-binding properties, thereby raising concern over its potential for human adaptation [6]. In 2023, a fatal mixed infection of H3N2 and H10N5 further underscored the emerging public health risks posed by H10 viruses [7]. These events highlight the importance of timely detection and surveillance of H5, H7, and H10 AIVs for early warning, outbreak containment, and preparedness against zoonotic threats.

Lateral flow immunoassays (LFAs) are widely used in clinical diagnostics owing to their simplicity, low cost, rapid turnaround time, and scalability [8,9]. However, the current gold standard for AIV detection, real-time reverse transcription polymerase chain reaction (RT-PCR), requires advanced laboratory infrastructure and trained personnel, making it unsuitable for point-of-care testing (POCT) during outbreak emergencies [10,11]. Thus, the development of highly sensitive, rapid, and field-deployable assays is urgently required. Upconversion nanoparticles (UCNPs) convert near-infrared excitation into visible or ultraviolet emission, offering sharp emission bands, high quantum yield and photostability [12,13]. For example, NaYF_4_:Yb,Er@NaYF_4_UCNPs enabled a smartphone-assisted LFA for hepatitis B virus detection with an LOD of 0.103 nM, markedly lower than the clinical threshold [14]. Similarly, NaYF_4_:Yb,Tm@NaYF_4_:15% Ca UCNPs achieved tenfold higher sensitivity than gold nanoparticle-based strips for AIV screening [15], and NaGdF_4_:Yb,Er@NaYF_4_ UCNPs combined with magnetic beads allowed HIV DNA detection at 0.0102 nM [16]. Overall, UCNPs effectively overcome background interference and scattering, making them highly promising fluorescent labels [17].

Recently, our group developed monoclonal antibodies (mAbs) specifically targeting the HA proteins of H5, H7, and H10 AIVs, which have demonstrated excellent diagnostic performance in immunoassays [18,19]. Building on this foundation, the present study aimed to construct a multiplex LFA platform employing UCNPs (NaYF_4_:Yb,Er@NaYF_4_:15%Ca) for the simultaneous detection of H5, H7, and H10 AIVs. The assay was further integrated with a smartphone-based application to enable quantitative analysis and real-time data sharing, thereby enhancing its applicability for field surveillance and outbreak response. This approach was designed to address current limitations of AIV detection methods by combining high sensitivity, specificity, and reproducibility with portability and user-friendliness.

## Materials and methods

### Monoclonal antibody and virus strains

**Figure 1.**
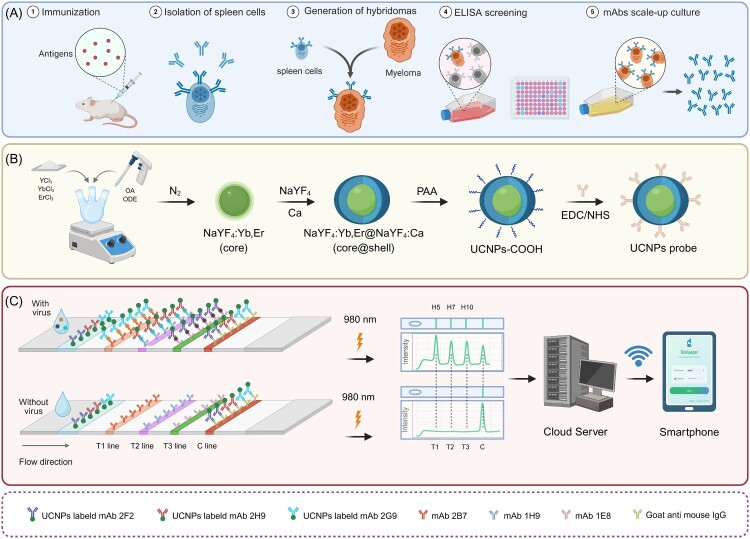
Schematic illustration of the core-shell UCNPs-LFA platform for simultaneous detection of H5, H7, and H10 AIVs. (A) Production of monoclonal antibodies using hybridoma technology. (B) Preparation of fluorescent probes based on core-shell UCNPs. (C) Application of the UCNPs-LFA strip in combination with a smartphone. T1, T2, and T3 correspond to the test lines for H5, H7, and H10 detection, respectively, while the C line serves as an internal control. This figure was created with BioRender.com.

The preparation of mAbs was performed using standard hybridoma technology. Briefly, mice were immunized with influenza virus HA antigens, after which spleen cells were fused with SP2/0 myeloma cells. Following ELISA screening and multiple rounds of subcloning, mAbs specific to H5 (2F2 and 2B7), H7 (1H9 and 2H9), and H10 (1E8 and 2G9) HA proteins were successfully generated [18,19] (Figure 1A). The fundamental properties of these mAbs are summarized in Table S1.

### Synthesis of core-shell UCNPs

NaYF4:Yb,Er core UCNPs were synthesized as previously described [20]. Briefly, 0.78 mmol YCl_3_·6H_2_O, 0.20 mmol YbCl_3_·6H_2_O, and 0.02 mmol ErCl_3_·6H_2_O were dissolved and dehydrated by heating in a three-necked flask. After water removal, 6 mL oleic acid and 15 mL 1-octadecene were added, and the mixture was heated to 150 °C. Following cooling to ambient temperature, a methanol solution containing 2.5 mmol NaOH and 4 mmol NH_4_F was introduced. The reaction mixture was then reheated to 110 °C for 10 min, followed by further heating under a nitrogen atmosphere to 300 °C for 90 min to promote crystal growth. After natural cooling to room temperature, the products were washed three times by centrifugation (10,000 rpm, 10 min) with sequential ethanol, cyclohexane, and ethanol. The final precipitate was redispersed in 20 mL cyclohexane.

NaYF_4_:Yb,Er@NaYF_4_:X%Ca (X = 0, 5, 10, 15, 20) core-shell UCNPs were prepared according to a modified protocol [21]. In brief, 0.5X% mmol CaCl_2_ and (0.5–0.5X%) mmol YCl_3_·6H_2_O were added to a three-necked flask, and the synthesis followed the same procedure as for core UCNPs, except that pre-formed core nanoparticles were introduced into the oleic acid/1-octadecene mixture after cooling to room temperature. Details of reagents and characterization instruments are provided in Table S2.

### Conjugation of antibodies with UCNPs

Core-shell UCNPs were purified by centrifugation (10,000 rpm, 10 min) three times with 0.05 mol/L HCl and ethanol to remove surface-bound oleic acid, as previously described [22]. The pellet was redispersed in ultrapure water and mixed with 1–5% polyacrylic acid (PAA, w/v) at a 2:1 volume ratio (UCNPs suspension: PAA). After 1 h of ultrasonic treatment, the nanoparticles were collected by centrifugation and redispersed in ultrapure water to obtain carboxyl-functionalised UCNPs (UCNPs-COOH).

For activation of carboxyl groups, 1 mg UCNPs-COOH was suspended in 1 mL MES buffer (0.05 mol/L, pH 6.5) containing 25 μL EDC (25 mg/mL) and 25 μL NHS (37.5 mg/mL) for 20 min. The activated UCNPs were then conjugated with three mAbs (2F2, 2H9, and 2G9) by incubation on a rotary shaker at room temperature for 2.5 h. The resulting antibody-UCNP conjugates were blocked with 1% bovine serum albumin (BSA, w/v) for 30 min and subsequently resuspended in 100 μL PBS (pH 7.4) containing 3% sucrose (w/v), 1% BSA (w/v), and 1% alginate (w/v). Probes were stored at 4 °C until use.

### Construction of the LFA strip

The structure of the multiplex LFA strip is shown in Figure 1. Each strip comprised a polyvinyl chloride (PVC) backing card, sample pad, conjugate pad, nitrocellulose (NC) membrane, and absorbent pad [23]. mAbs2B7 (0.8 mg/mL), 1H9 (0.8 mg/mL), and 1E8 (1.0 mg/mL), together with goat anti-mouse IgG (1.0 mg/mL), were dispensed onto the NC membrane at a rate of 1.0 μL/cm to generate the H5 (T1), H7 (T2), H10 (T3), and control (C) lines, respectively. The NC membrane was dried at 36 °C for 12 h. The conjugate and absorbent pads were pretreated by immersion in PBS (pH 7.4) containing 3% sucrose, 1% bovine serum albumin (BSA), and 1% sodium alginate (all w/v). Subsequently, the sample pad, conjugate pad, NC membrane, and absorbent pad were assembled sequentially onto the PVC backing card. The assembled sheets were cut into strips 3.7 mm in width and stored in a dry environment until use.

### Performance evaluation of the LFA strip

#### Sensitivity

Two-fold serial dilutions of allantoic fluids containing H5, H7, and H10 subtype viruses were prepared, with titres ranging from 2^5^ to 2^−7^ HAU in PBST. In parallel, purified HA proteins of each subtype were diluted across a concentration range of 128 to 0.0078 ng/mL. A 50 μL aliquot of each sample was applied to the test strip and incubated at room temperature for 8 min. Fluorescence intensity was measured under 980 nm excitation using a fluorescence scanner, and the ratio of the peak area at the test line to that at the control line (T/C value) was calculated. Calibration curves for H5, H7, and H10 were constructed using the logarithm of the standard solution concentration (log_10_C) as the X-axis and the logarithm of the T/C value (log_10_T/C) as the Y-axis.

The cut-off value was defined as the mean T/C value of ten blank PBST samples minus three times the standard deviation (mean − 3SD) [24]. A sample was interpreted as negative if its T/C value fell below the threshold and positive if it exceeded it. The lowest concentration yielding a T/C value above the cut-off was considered the limit of detection (LOD) of the LFA strip. To enable comparison of sensitivity with other antigen detection assays, the TCID_50_/mL of the allantoic fluid viruses used in this study were determined and correlated with their HAU. For the LOD determination of the LFA, each virus was diluted with negative respiratory specimen matrix (throat swab samples) to obtain concentrations corresponding to the LOD expressed in HAU. Virus was serially tenfold diluted in infection medium and inoculated onto confluent MDCK cell monolayers in 96-well plates. Plates were incubated at 37 °C with 5% CO_2_ and observed daily for cytopathic effect (CPE) for up to five days. Wells showing CPE were recorded as positive, and TCID_50_/mL was calculated using the Reed-Muench method. LOD was defined as the lowest virus concentration at which ≥95% of replicates tested positive under these assay conditions.

#### Specificity

To evaluate the specificity of the UCNPs-based multiplex LFA, purified allantoic fluids from H5, H7, and H10 subtype AIVs were tested alongside a panel of non-target AIV subtypes and respiratory viruses (Table 1). The T/C values obtained from the test lines were substituted into the standard calibration curves for each HA subtype. Following the method of a previous study [25], equivalent titres (eH5, eH7, and eH10) were calculated using the fitted equations. Cross-reactivity was then determined by comparing the T/C-derived equivalent titre of each non-target sample (eX) with that of the corresponding target virus at the reference concentration (0.5 HAU), according to the following formula:

$$\mathrm{Cross}-\mathrm{reactivity}\;(\text{\%})=\frac{\mathrm{eX}}{0.5}\times 100\text{\%}$$

**Table 1.** Respiratory viruses tested in this study.

No.	Virus	Strain name
1	H5N1(2.3.2)	A/goose/Zhejiang/727098/2014
2	H5N1(2.3.4.4 g, Re^a^ 8)	A/chicken/Guizhou/4/2013
3	H5N1(2.3.4.4b, Re)	A/Texas/37/2024
4	H5N2 (2.3.4.4c)	A/chicken/Zhejiang/514135/2015
5	H5N2(2.3.4.4b)	A/duck/Zhejiang/6DK19/2013
6	H5N6 (2.3.4.4b)	A/duck/Zhejiang/6D2/2013
7	H5N6 (2.3.4.4 h, Re 11)	A/duck/Guizhou/S4184/2017
8	H5N6 (2.3.4.4 h, Re 13)	A/duck/Fujian/S1424/2020
9	H5N8 (2.3.4.4b, Re 14)	A/whooper swan/Shanxi/4–1/2020
10	H5N8 (2.3.4.4b)	A/duck/Zhejiang/W24/2013
11	H7N3	A/duck/Zhejiang/DK16/2013
12	H7N3	A/chicken/Zhejiang/92752/2015
13	H7N7	A/chicken/Jiangxi/C25/2014
14	H7N9	A/Zhejiang/DTID-ZJU01/2013
15	H7N9 (highly pathogenic virus)	A/Guangdong/HP001/2017
16	H7N9 (highly pathogenic virus, Re3)	A/chicken/Inner Mongolia/SD010/2019
17	H7N9 (highly pathogenic virus, Re4)	A/chicken/Yunnan/SD024/2021
18	H7N9	A/chicken/Zhejiang/1128/2023
19	H10N2	A/duck/Zhejiang/6D20/2013
20	H10N3	A/chicken/Zhejiang/516100/2017
21	H10N3	A/Zhejiang/CDK/2022
22	H10N5	A/Zhejiang/CNIC-ZJU01/2023
23	H10N7	A/chicken/Zhejiang/2CP8/2014
24	H10N8	A/chicken/Zhejiang/102615/2016
25	H1N1 (pdm09)	A/California/07/2009
26	H1N2	A/duck/Zhejiang/D1/2013
27	H2N8	A/duck/Zhejiang/6D10/2013
28	H3N2	A/duck/Zhejiang/4613/2013
29	H3N2	A/Texas/50/2012(H3N2)
30	H4N2	A/chicken/Zhejiang/727145/2014
31	H4N6	A/duck/Zhejiang/409/2013
32	H6N1	A/chicken/Zhejiang/1664/2017
33	H6N6	A/chicken/Zhejiang/727028/2014
34	H9N2	A/chicken/Zhejiang/221/2016
35	H9N2	A/chicken/Zhejiang/61174/2017
36	H11N3	A/duck/Zhejiang/727D2/2013
37	H11N9	A/duck/Zhejiang/71750/2013
38	Marek’s disease virus (MDV)	FC-126
39	Avian pox virus (APV)	Quail-Adapted strain
40	Avian paramyxovirus-4 (APV-4)	ZJ-1
41	Infectious bronchitis virus (IBV)	H120
42	Infectious bursal disease virus (IBDV)	NF8
43	Newcastle disease virus (NDV)	La Sota
44	Infectious laryngotracheitis virus (ILV)	K317

Note: ^a^Re, the vaccine strains which contained surface two genes (HA and NA) from H5 virus, and six internal genes from A/Puerto Rico/8/1934 (H1N1) (PR8) virus.

#### Reproducibility

The reproducibility of the LFA strip was assessed by repeated assays, with results expressed as the coefficient of variation (CV). Ten strips, randomly selected from both the same and different production batches, were tested using HA protein samples at concentrations of 64, 4, 0.25, and 0 ng/mL. In addition, we also used virus allantoic fluid close to the LODs for reproducibility verification. The corresponding mean values, standard deviations, and CVs were calculated to evaluate signal consistency.

### Detection of actual samples

The applicability of the LFA strips for real sample detection was evaluated using a total of 135 avian specimens, comprising 104 cloacal swabs and 31 faecal samples collected from poultry in Zhejiang Province, Eastern China, between 2013 and 2023. Of these, 15 samples were positive for H5 virus, 9 were positive for H7 virus, and 2 were positive for H10 virus, as determined by RT-PCR [26]. In addition, 125 human clinical samples, including 76 throat swabs and 49 sputum samples collected between 2013 and 2020, were analyzed. Among these, 17 were positive for H1N1 influenza virus, 13 for H3N2 influenza virus, and 3 for H7N9 influenza virus. A further 7 samples tested positive for influenza B virus, 2 for rhinoviruses, 4 for adenoviruses, 2 for respiratory syncytial viruses, and 5 for SARS-CoV-2 [27,28]. To ensure consistency with the RT-PCR reference method during the comparative evaluation, the following sample pretreatment steps were used. Cloacal and throat swabs, collected in viral transport media, were vortexed to elute viral particles and then centrifuged at 3,000 × g for 5 min to obtain a clarified supernatant. Fecal samples were prepared as a 1:10 (w/v) suspension in PBS, which was thoroughly vortexed and clarified by high-speed centrifugation at 12,000 × g for 10 min. For the viscous sputum samples, an equal volume of Sputasol was added for liquefaction, followed by incubation and vortexing, prior to centrifugation at 3,000 × g for 10 min. The resulting supernatant from all sample types was used for the LFA strips test. Finally, 30 RT-PCR-negative throat swabs were spiked with H5, H7, or H10 allantoic fluid to achieve a final concentration of 0.5 HAU, thereby simulating authentic clinical samples.

### Design of the intelligent LFA platform

To meet the requirements of POCT, a sample treatment buffer was prepared according to a previous study [29] with slight modifications. The buffer consisted of 0.4% polyvinylpyrrolidone (PVP), 0.7% surfactant 10 G, 0.1% gelatin, and 0.1% BSA, dissolved in 1×PBS. For sample preparation, the specimen (e.g. a throat swab) was placed directly into the buffer, manually shaken, and left to stand at room temperature for 5 min. The supernatant was then collected for testing. Fluorescence signals from the LFA strips were scanned using a fluorescence scanner, and the resulting data were uploaded to a cloud server. Peak areas corresponding to the T1, T2, T3, and C lines were calculated, and the ratios T1/C, T2/C, and T3/C were obtained. All data analyses within the intelligent LFA platform were based on T/C ratios and spectral profiles. To streamline data processing and enable practical sample testing, an intelligent system was developed using JavaScript, incorporating both web-based and mobile interfaces [20]. The system architecture is shown in Figure S1.

## Results

### Characterizations of NaYF_4_:Yb,Er@NaYF_4_:Cacore-shell UCNPs

**Figure 2.**
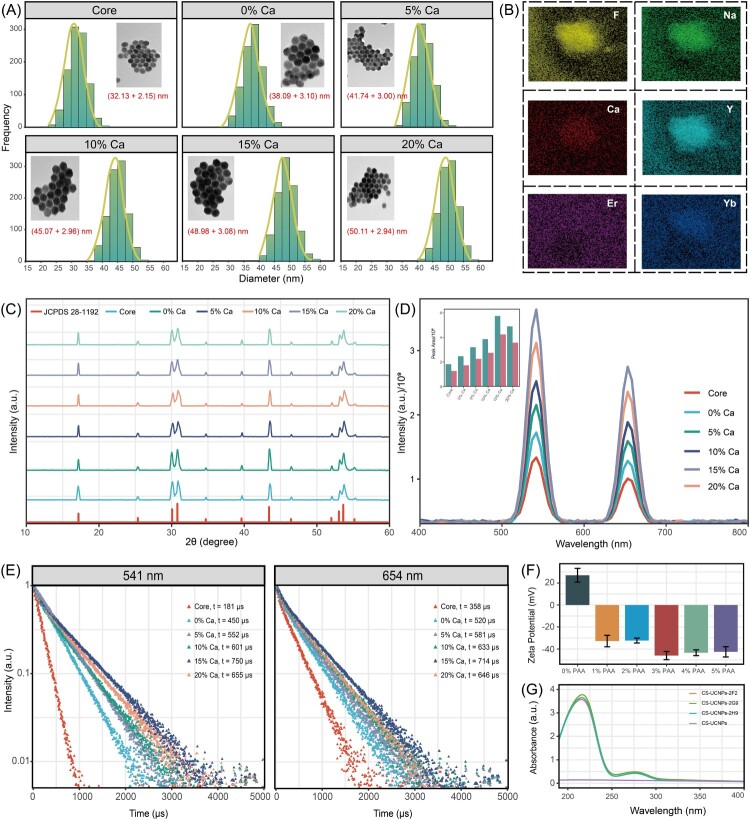
Characterization of core-shell UCNPs. (A) TEM images and size distribution at different Ca^2+^ concentrations. (B) Elemental analysis by EDS. (C) XRD patterns. (D) Upconversion emission spectra. (E) Fluorescence lifetime profiles. (F) Zeta potential of carboxylated UCNPs. (G) UV-Vis spectra of fluorescent probes.

A series of core-shell UCNPs with Ca^2+^ doping ratios ranging from 0 to 15% were synthesized. As shown in Figure 2A, all six groups exhibited a uniform hexagonal phase structure with smooth surfaces, well-defined shapes, and homogeneous dimensions. UCNPs doped with 10% and 15% Ca^2+^ displayed sharper crystal boundaries and more distinct hexagonal morphology. Statistical analysis of TEM images revealed that the average diameter of the core UCNPs was approximately 32.13 ± 2.15 nm, whereas the core-shell UCNPs ranged from 38.09 to 50.11 nm.

Elemental composition analysis (Figure 2B) demonstrated that the core-shell UCNPs were composed of Na, Y, F, Yb, Er, and Ca. The Na, Y, F, Yb, and Er elements were evenly distributed, while Yb and Ca showed relatively sparse distributions due to their lower concentrations. Elemental mapping confirmed the successful incorporation of Yb^3+^ and Ca^2+^ ions into the NaYF_4_ lattice, forming a solid solution without detectable impurities.

XRD patterns (Figure 2C) further confirmed that the diffraction peak positions and intensities of all six groups were consistent with the standard hexagonal phase (β-NaYF_4_, JCPDS No. 28–1192), indicating identical growth orientations and a single lattice phase without coexistence. Increasing Ca^2+^ doping enhanced peak intensity, suggesting improved crystallinity and phase purity. These findings are consistent with TEM observations, confirming that both core and core-shell UCNPs possessed a pure hexagonal phase with high crystallinity.

To examine the effect of Ca^2+^ content on upconversion luminescence, emission spectra were recorded under 980 nm continuous-wave laser excitation (Figure 2D). Core-shell UCNPs exhibited strong dual-band emissions in the green (541 nm) and red (654 nm) regions, with markedly enhanced fluorescence intensities compared with core counterparts. Emission intensity increased with Ca^2+^ doping up to 15% before declining at 20%, indicating optimal luminescence efficiency at 15% Ca^2+^.

Transient fluorescence lifetime measurements (Figure 2E) supported these findings. Core UCNPs displayed the shortest lifetimes (181 μs at 541 nm; 358 μs at 654 nm), while shell-coated UCNPs doped with 15% Ca^2+^ showed the longest lifetimes (750 and 714 μs, respectively). Further increasing the Ca^2+^ content to 20% reduced lifetimes (655 and 646 μs), consistent with diminished emission efficiency. Based on these results, 15% Ca^2+^-doped core-shell UCNPs were selected for subsequent experiments.

### Conjugation of antibodies with UCNPs

To assess the effect of different PAA concentrations on the carboxylation efficiency of UCNPs during surface modification, the Zeta potentials of the particles were measured before and after functionalisation. As shown in Figure 2F, treatment with 1% PAA reduced the surface Zeta potential from +27.09 mV to −32.76 mV. When the PAA concentration was increased to 3% or higher, the potential stabilized within the range of −42.59 to −45.96 mV. FTIR spectra of PAA-modified UCNPs further confirmed successful carboxylation, displaying characteristic absorption bands (Figure S2A). The peak at 2840 cm^−1^ was attributed to C–H stretching vibrations of –CH_2_– and –CH_3_ groups, while strong absorptions at 1604 and 1405 cm^−1^ corresponded to the asymmetric and symmetric stretching of carboxylate (–COO^−^) groups.

Following antibody conjugation, UCNPs exhibited distinct ultraviolet absorption peaks at approximately 220 and 280 nm, with intensities exceeding 0.42 and 3.60, respectively (Figure 2G). In contrast, unmodified UCNPs showed no prominent ultraviolet peaks, confirming the successful attachment of antibodies to the UCNPs surface. The conjugation efficiency of 3% PAA-modified UCNPs with mAbs was further quantified using a BCA protein assay to measure residual protein in the post-conjugation supernatant. Binding efficiencies for mAbs 2F2, 2G9, and 2H9 were calculated to be 74.54%, 81.50%, and 77.22%, respectively (Figure S2B).

### Performance evaluation of the LFA strip

**Figure 3.**
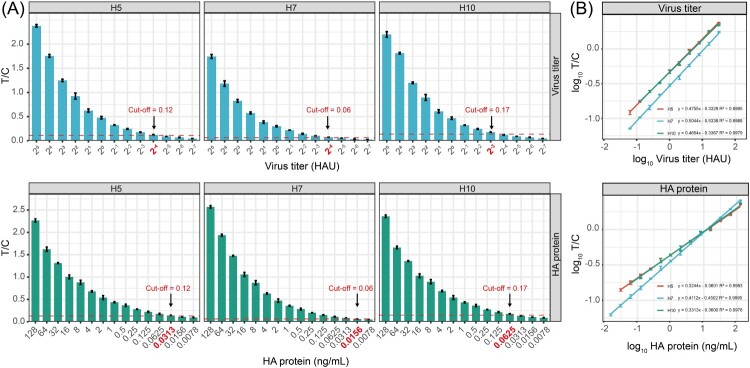
Sensitivity of UCNPs-LFA strips for detecting H5, H7, and H10 AIVs in allantoic fluid and purified HA protein. (A) Detection of allantoic fluid at viral titres ranging from 2^5^ to 2^−7^ HAU and HA protein concentrations from 128 to 0.0078 ng/mL. (B) Standard curves for viral titres and HA protein concentrations.

As shown in Figure 3A, the cut-off values for H5, H7, and H10 subtypes were 0.12, 0.06, and 0.17, respectively. For viral titre detection (Figure 3B), the linear regression equations were y = 0.4755x − 0.3328 (H5), y = 0.5044x − 0.5338 (H7), and y = 0.4654x − 0.3367 (H10), each demonstrating strong linearity within the ranges of 2^−4^ to 2^5^, 2^−4^ to 2^5^, and 2^−3^ to 2^5^ HAU, respectively. For purified HA protein detection, the regression equations were y = 0.3244x − 0.3601 (H5), y = 0.4112x − 0.4502 (H7), and y = 0.3313x − 0.3600 (H10), with good linearity across the ranges of 0.0313–128 ng/mL, 0.0156–128 ng/mL, and 0.0625–120 ng/mL, respectively. Consequently, the LODs of the LFA strip for H5, H7, and H10 subtype AIVs were 2^−4^, 2^−4^, and 2^−3^ HAU, and 0.0313, 0.0156, and 0.0625 ng/mL, respectively. To enable comparison with other antigen detection assays, the HAU-based sensitivities were converted to infectious titres (Table S3). The results showed that the LODs corresponded to approximately 2.51 × 10^2^, 1.00 × 10^2^, and 3.16 × 10^2^ TCID_50_/mL for H5, H7 and H10, respectively.

**Figure 4.**
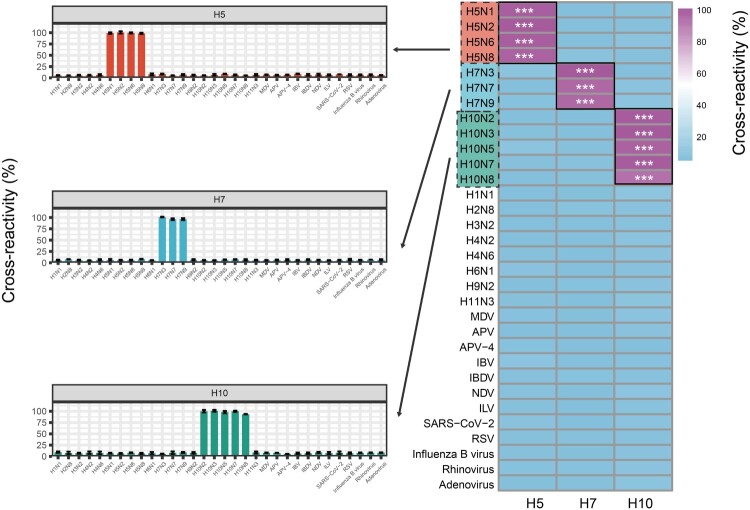
Cross-reactivity analysis of UCNPs-LFA strips in detecting H5, H7, and H10 subtype AIVs and other viruses. ***P < 0.001.

Specificity was further assessed against multiple AIV subtypes and other respiratory pathogens (Figure 4). The LFA strips demonstrated exceptionally high specificity for H5, H7, and H10 subtypes, with detection rates exceeding 95% (*P* < 0.001), which were significantly higher than those for non-target viruses. For the latter, cross-reactivity remained below 10% with no statistically significant differences (*P* > 0.05). The LFA exhibited excellent specificity for H5, H7, and H10 subtype AIVs, as all target viruses (n = 12) yielded T/C values above cut-off thresholds, while all non-target virus (n = 20) produced values below the cut-offs (Table S4). These findings confirm that the developed LFA strips reliably distinguish target subtypes from other respiratory pathogens.

Reproducibility was evaluated using strips from both the same and different production batches (Table 2). No significant variation in T/C values was observed between replicates, and both intra- and inter-batch CVs were below 6%. In the supplemental reproducibility study conducted near the LOD (Table S5), intra- and inter-assay CVs remained below 4% for all three targets (H5, H7, and H10). These results demonstrate that the LFA strips exhibit high precision and stable performance.

**Table 2.** Reproducibility of UCNPs-LFA strips for detection of H5, H7, and H10 subtype AIVs.

		0 ng/mL			0.25 ng/mL			4 ng/mL			64 ng/mL		
		T1/C	T2/C	T3/C	T1/C	T2/C	T3/C	T1/C	T2/C	T3/C	T1/C	T2/C	T3/C
Intra-assay (n = 10)	Mean	0.1233	0.0634	0.1744	0.2802	0.2159	0.2745	0.6677	0.6406	0.686	1.2627	1.3532	1.2648
	SD	0.0026	0.002	0.0028	0.0056	0.0102	0.0035	0.0277	0.0269	0.021	0.042	0.0343	0.0342
	CV (%)	2.081	3.1308	1.6254	2.011	4.7175	1.2836	4.1422	4.2061	3.0621	3.3273	2.5329	2.7035
Inter-assay (n = 10)	Mean	0.1239	0.6481	0.1742	0.2737	0.216	0.277	0.6803	0.6251	0.6796	1.2534	1.3322	1.2312
	SD	0.0021	0.0371	0.0028	0.0034	0.0094	0.0091	0.0226	0.0177	0.0301	0.029	0.0775	0.0431
	CV (%)	1.6949	5.7273	1.593	1.2351	4.3338	3.2842	3.3158	2.8302	4.4305	2.3113	5.8144	3.5023

### Actual sample analysis

As summarized in Table 3, the LFA strips showed complete concordance with real-time PCR results across all tested samples. Among the 135 avian samples, 15 were positive for H5, 9 for H7, and 2 for H10, with identical outcomes obtained by both methods. Of the 125 human samples, 3 were positive for H7, while none were positive for H5 or H10, again demonstrating full agreement between the LFA assay and PCR. In the spiked sample evaluation, all swabs supplemented with H5, H7, or H10 HA allantoic fluid were correctly identified by the LFA strips, with no cross-detection of non-target subtypes. These findings confirm that the developed LFA strip provides accurate, subtype-specific detection of H5, H7, and H10 AIVs, showing excellent consistency with RT-PCR in both naturally infected and artificially spiked samples.

**Table 3.** Detection performance of UCNPs-LFA strips compared with real-time PCR for avian, human, and spiked samples.

Sample type	Number	LFA result (*P*/N^a^)			real-time PCR result (*P*/N)		
		H5	H7	H10	H5	H7	H10
Avian samples	135	15/120	9/126	2/135	15/120	9/126	2/135
Human samples	125	0/125	3/122	0/125	0/125	3/122	0/125
H5-spiked samples	10	10/0	0/10	0/10	10/0	0/10	0/10
H7-spiked samples	10	0/10	10/0	0/10	0/10	10/0	0/10
H10-spiked samples	10	0/10	0/10	10/0	0/10	0/10	10/0

Note: ^a^P/N, number of positives samples/ number of negatives samples.

### Application of the intelligent system

**Figure 5.**
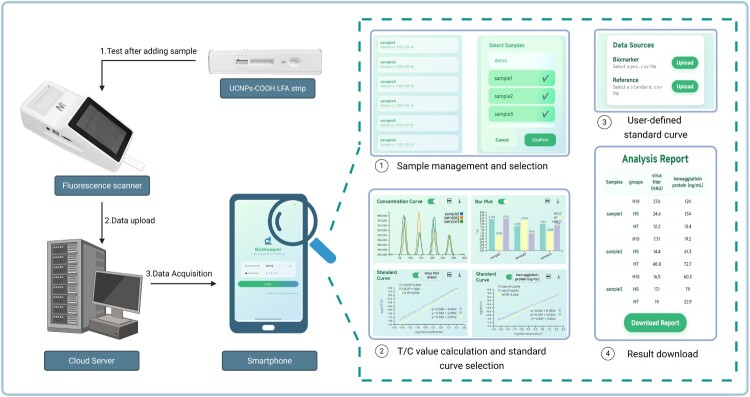
Schematic illustration of the smartphone-assisted UCNPs-LFA rapid detection platform.

In this study, a user-friendly intelligent application system was developed to enable rapid quantitative detection of H5, H7, and H10 subtype AIVs using the core-shell UCNPs-LFA platform. The system supports on-site measurements with data synchronized in real time to remote databases. It comprises two main components: a cloud server and a mobile client. Once raw data from field testing are uploaded to the server, the corresponding sample records are updated automatically. The mobile client incorporates multiple functions, including user account management, sample tracking, graphical data visualization, automated T/C ratio calculation, generation of customizable standard curves, and export of detection results. Users can switch flexibly between single-virus and multi-virus detection modes according to specific testing needs. The operational workflow consists of the following steps: (1) user login; (2) database synchronization and sample selection; (3) specification of the target subtype AIV; (4) calculation of the sample’s T/C ratio; (5) selection of the corresponding standard curve; (6) determination of viral titre or HA protein concentration; and (7) export of the detection report. A schematic of the system is presented in Figure 5.

## Discussion

The incorporation of Ca²^+^ markedly influenced the structural and optical properties of the synthesized UCNPs. Calcium doping is known to enhance energy transfer efficiency between sensitizer and activator ions, thereby increasing upconversion luminescence intensity [30,31]. The observed enlargement in particle diameter following shell coating is consistent with the addition of an epitaxial NaYF_4_:Ca layer, which not only increased overall size but also provided a protective barrier that suppressed surface-related quenching processes [32]. The well-defined hexagonal phase morphology observed in TEM is advantageous, as hexagonal-phase UCNPs possess lower phonon energy and higher upconversion efficiency than the cubic phase, in agreement with previous reports [33]. XRD analysis further demonstrated enhanced diffraction peak intensity and sharper hexagonal-phase features upon Ca^2+^ incorporation, suggesting improved crystallinity and phase purity, which corroborates the TEM observations [34]. Emission spectra showed that luminescence intensity increased with Ca^2+^ doping up to 15%, but declined at 20%. This reduction is likely due to excessive Ca^2+^ introduction, which may generate crystal defects or lattice strain, thereby facilitating non-radiative pathways and concentration quenching [35]. A similar trend was observed in fluorescence lifetime measurements, where lifetimes were prolonged up to 15% doping owing to reduced surface quenching and minimized cross-relaxation. However, at 20% Ca^2+^, enhanced non-radiative relaxation and defect-related energy dissipation likely shortened the lifetime [36]. These findings are consistent with previous reports defining optimal doping ranges for maximizing UCNPs luminescence efficiency [37].

In this study, PAA surface modification effectively reduced the Zeta potential of UCNPs from +27.09 mV to below −40 mV, thereby ensuring high stability of the resulting UCNPs-COOH in aqueous media [38]. Compared with silanisation or ligand-exchange methods, PAA coating introduced abundant –COOH groups in a simple, mild, and reproducible manner, as confirmed by FTIR peaks at 1604 cm^−1^ and 1405^−1^ cm, corresponding to asymmetric and symmetric –COO^−^ stretching vibrations, respectively [39]. Ultraviolet-visible (UV-Vis) detection of characteristic absorption peaks at 220 and 280 nm further confirmed antibody conjugation, although such spectral analysis does not provide quantitative information on coupling efficiency. To address this, residual protein content was measured using a BCA assay. The conjugation efficiencies of mAbs 2F2, 2G9, and 2H9 were all above 70%, indicating efficient functionalisation and suitability of the probes for high-sensitivity bioassays.

The UCNPs-based LFA strips developed in this study demonstrated high analytical sensitivity, with LODs as low as 2^−4^ HAU for H5 and H7 and 2^−3^ HAU for H10, as well as sub-nanogram levels for purified HA proteins. The UCNPs-based LFA strips developed in this study demonstrated high analytical sensitivity, with LODs of approximately 2.51 × 10^2^, 1.00 × 10^2^, and 3.16 × 10^2^ TCID_50_/mL for H5, H7 and H10, respectively. This level of sensitivity has been reported in the literature as sufficient for detecting viral shedding in early infection [40,41], suggesting the potential of our assay for early-stage AIVs detection. Compared with conventional colloidal gold LFAs, the upconversion luminescence readout of UCNPs reduces background autofluorescence [42], thereby improving trace-level detection. Although quantum dot (QD)-based LFAs have achieved comparable sensitivity, such as the use of chiral MoS_2_ QDs for detecting H5N1 with an LOD of 80.92 pg/mL [43], UCNPs offer distinct advantages by avoiding photobleaching and reducing concerns over heavy metal toxicity, thus providing improved biocompatibility [44]. Similarly, time-resolved fluorescence microsphere-based LFAs have been reported to enable quantitative detection of African swine fever virus within a range of 0.24–500 ng/mL [45], indicating that UCNPs-based assays can achieve comparable sensitivity.

The strips also exhibited excellent specificity, with cross-reactivity rates exceeding 95% for the target subtypes and remaining below 10% for non-target respiratory pathogens. This high specificity is attributed to the strong affinity and conserved epitopes of the mAbs used, with binding sites showing more than 84% conservation, ensuring accurate recognition of the target antigens and enhancing assay performance. In terms of precision, both intra- and inter-batch CVs were below 6%, indicating robust reproducibility. Such consistency is critical for large-scale deployment, as batch-to-batch variation can compromise reliability in clinical and field applications [46].

Compared with colloidal gold LFAs, UCNPs-based strips also maintain stable luminescence during long-term storage due to the chemical stability of the nanomaterial coating [47]. By contrast, QD-based assays are prone to photodegradation, while time-resolved microspheres are often associated with higher material and operational costs [48], which may limit their widespread use. Furthermore, UCNPs can be synthesized and surface-modified using relatively straightforward procedures, offering better control over particle properties while remaining amenable to large-scale production. Collectively, the UCNPs-LFA platform presented here achieves a favourable balance of sensitivity, specificity, and reproducibility, making it well suited for on-site screening of multiple AIV subtypes.

Evaluation of the UCNPs-LFA using a broad panel of avian, human, and spiked samples demonstrated its robust applicability across diverse testing scenarios. The assay showed complete concordance with RT-PCR results for all 290 samples, underscoring its high reliability and diagnostic accuracy. This strong agreement validates the UCNPs-LFA as a dependable alternative for rapid AIV subtype identification, particularly in resource-limited settings where PCR facilities may be unavailable. Importantly, its ability to detect H5, H7, and H10 subtypes without cross-reactivity addresses the critical need for subtype-specific tools in both avian health monitoring and zoonotic risk assessment, thereby supporting public health efforts to prevent and control outbreaks [49,50]. Integration of the UCNPs-LFA with a smartphone-based intelligent system further enhanced its utility by enabling rapid, on-site quantitative detection and real-time remote data synchronization, delivering results within 10 min of sample loading. Comparable smartphone-assisted LFAs have been reported for infectious disease diagnostics. For example, a method combining loop-mediated isothermal amplification with CRISPR/Cas12a enabled SARS-CoV-2 detection with accurate results obtained within 40 min on a smartphone platform [51]. In another study, a graphene-based sensor was implemented on a mobile device for monkeypox virus detection, achieving an LOD of 0.75 PFU/mL [52]. These studies illustrate the potential of mobile platforms to improve accessibility and timeliness of diagnostics. By incorporating automated T/C ratio calculation, standard curve matching, and instant cloud-based data sharing, the present UCNPs-LFA system aligns with the growing trend towards digitalized and networked diagnostics. This integration provides a practical solution for both field surveillance and decentralized clinical testing, bridging laboratory precision with point-of-care accessibility.

## Conclusion

In this study, a smartphone-assisted immunochromatographic assay was developed for the simultaneous detection of H5, H7, and H10 subtype AIVs using core-shell UCNPs. The UCNPs-LFA platform demonstrated several key advantages, including high analytical sensitivity, strong specificity, operational reliability, and user-friendliness. The LODs for HA proteins and viral titres of H5, H7, and H10 were exceptionally low, confirming its suitability for detecting AIVs even at low viral loads. The assay also showed excellent specificity, with no cross-reactivity to other respiratory pathogens, and results from actual samples were in complete concordance with RT-PCR. By enabling rapid, accurate, and subtype-specific identification, this platform offers a dependable tool for early diagnosis and cross-regional surveillance of major avian diseases. Its integration with smartphone technology further enhances accessibility and practicality in field settings. Collectively, the UCNP-LFA system holds significant potential to strengthen outbreak preparedness and contribute to the prevention and control of zoonotic influenza, thereby supporting global public health security.

## Supplementary Material

Supplementary Tables.docx

Figure S2.jpg

Figure S1.jpg

## Data Availability

The datasets used and/or analyzed during the current study are available from the corresponding author upon reasonable request.
