# Supplementary material for: Smartphone-assisted upconversion nanoparticle assay for rapid multiplex detection of H5, H7, and H10 avian influenza viruses
Source: Emerg Microbes Infect. 2025 Dec 9;15(1):2602315. doi: 10.1080/22221751.2025.2602315 (PMC12777912; doi:10.1080/22221751.2025.2602315)
Supplement: Supplementary Tables.docx [file TEMI_A_2602315_SM9129.docx]

**Table S1** Monoclonal antibodies used in this study and their characteristics.

|  | **Name** | **Affinity (µg/mL)** | **Isotype** | |  | **Conserved amino acid sites** | |  | **Heavy chain** | |  | **Light chain** | |
| --- | --- | --- | --- | --- | --- | --- | --- | --- | --- | --- | --- | --- | --- |
|  |  |  | **Subclass** | **Type** |  | **Residue** | **Epitope conservation (%)** |  | **V-GENE and allele** | **CDR3^a^** |  | **V-GENE and allele** | **CDR3** |
| H5 | 2F2 | 6×10^−6^ | IgG2a | k |  | R239 | 84.31 |  | Musmus IGHV2-6-7*01 F | ARDGGYGYGFAY |  | Musmus IGKV12-46*01F | QHFWGTPLT |
|  | 2B7 | 9×10^−5^ | IgG2a | k |  | A172 | 94.78 |  | Musmus IGHV1-18*01 F | ARSDDHDAWFAY |  | Musmus IGKV1-135*01 F | WQGTHFPQT |
| H7 | 1H9 | 1×10^−2^ | IgG1a | k |  | G132 | 97.7 |  | Musmus IGHV9-3-1*01F | ARNPYNYRYDGAWFVY |  | Musmus IGKV5-48*01 F | QQSDSWPLT |
|  | 2H9 | 5×10^−2^ | IgG1a | k |  | N167 | 99.6 |  | Musmus IGHV14-3*02F | ARVYGGKFDV |  | Musmus IGKV14-100*01 F | VQYAQWPYT |
| H10 | 1E8 | 4×10^−4^ | IgG2b | k |  | K165 | 99.78 |  | Musmus IGHV8-12*02 F | ARSPPTDYGSSWGVMDY |  | Musmus IGKV4-59*01 F | QQWSSNPLT |
|  | 2G9 | 2×10^−4^ | IgG2a | k |  | N170 | 99.89 |  | Musmus IGHV9-3-1*01 F | ARGYDYAEGYFAMDY |  | Musmus IGKV8-30*01 F | QQYYSNYT |

Note: ^a^CDR, Complementarity-determining region.

**Table S2** Reagents, instruments, and characterizations used in this study.

| **Reagents** |  | YCl_3_·6H_2_O (99.9%), YbCl_3_·6H_2_O (99.9%), ErCl_3_·6H_2_O (99.9%), CaCl_2_ (99.9%), polyacrylic acid (PAA, 98%), bovine serum albumin (BSA, 98%), N-hydroxysuccinimide (NHS, 98%), N-(3-dimethylaminopropyl)-N′-ethylcarbodiimide hydrochloride (EDC, 98%), and morpholineethanesulfonic acid (MES, 99%) were purchased from Sigma-Aldrich (Shanghai, China). Ammonium fluoride (NH_4_F, 96%), oleic acid (90%), 1-octadecene (90%), methanol (AR), ethanol (AR), cyclohexane (AR), sodium hydroxide (NaOH, AR), and hydrochloric acid (HCl, 10 mol/L) were obtained from Macklin Reagent Co., Ltd. (Shanghai, China). |
| --- | --- | --- |
| **Instruments**  **and Characterizations** |  | The morphology and particle size of the upconversion nanoparticles (UCNPs) were observed and measured using transmission electron microscopy (TEM, Tecnai G2 F20, FEI, USA). The elemental composition of the UCNPs was analyzed by scanning electron microscopy (SEM, SU8230, Hitachi High-Tech, Japan) equipped with an energy-dispersive spectrometer (EDS, Oxford Instruments, UK). The crystalline structure of the nanoparticles was examined through X-ray diffraction (XRD, BRUKER., Germany) analysis. The emission spectra and fluorescence lifetimes of the UCNPs under 980 nm excitation were recorded using a fluorescence spectrometer (FLS1000, Edinburgh Instruments, UK) capable of both steady-state and time-resolved measurements. The surface potential of polyacrylic acid (PAA)-modified UCNPs was determined using aZetasizer Nano ZS (Malvern Instruments, UK). The changes in surface functional groups of the dual-conversion nanoparticles were analyzed using a Fourier-transform infrared (FTIR) spectrometer (Nicolet iS50, Thermo Fisher Scientific, USA). The absorbance of antibody-conjugated nanoparticles was measured with a UV-Vis spectrophotometer (Cary 60, Agilent Technologies, USA). The conjugation efficiency of antibodies to the UCNPs was evaluated using a BCA protein assay kit (Thermo Scientific, USA), following the manufacturer’s instructions Test strip test results were from 980 nm excitation light fluorescence scanner (Niutai Biotechnology Co., Ltd., Hangzhou, China). |

**Table S3** Correlation between HAU and TCID_50_/mL of allantoic fluid virus used for LFA LOD evaluation.

| Virus subtype | HAU | TCID_50_/mL |
| --- | --- | --- |
| H5 | 2^-4^ | 2.51×10^2^ |
| H7 | 2^-4^ | 1.00×10^2^ |
| H10 | 2^-3^ | 3.16×10^2^ |

**Table S4** Specificity evaluation of the LFA for target and non-target viruses.

| **Virus subtype / pathogen** | **Strain name** | **T_1_/C** | **T_2_/C** | **T_3_/C** | **Cut-off value** | **Result** |
| --- | --- | --- | --- | --- | --- | --- |
| H5N1 | A/Texas/37/2024 | 0.3347 | 0.0408 | 0.0906 | (0.12/0.06/0.17) | + |
| H5N2 | A/chicken/Zhejiang/514135/2015 | 0.3319 | 0.0406 | 0.0866 | (0.12/0.06/0.17) | + |
| H5N6 | A/duck/Fujian/S1424/2020 | 0.3324 | 0.0423 | 0.1091 | (0.12/0.06/0.17) | + |
| H5N8 | A/whooper swan/Shanxi/4-1/2020 | 0.3345 | 0.0588 | 0.1045 | (0.12/0.06/0.17) | + |
| H7N3 | A/chicken/Zhejiang/92752/2015 | 0.1066 | 0.2071 | 0.087 | (0.12/0.06/0.17) | + |
| H7N7 | A/chicken/Jiangxi/C25/2014 | 0.0843 | 0.2012 | 0.0856 | (0.12/0.06/0.17) | + |
| H7N9 | A/chicken/Zhejiang/1128/2023 | 0.0922 | 0.2015 | 0.0992 | (0.12/0.06/0.17) | + |
| H10N2 | A/duck/Zhejiang/6D20/2013 | 0.0732 | 0.0468 | 0.3286 | (0.12/0.06/0.17) | + |
| H10N3 | A/Zhejiang/CDK/2022 | 0.0866 | 0.0406 | 0.3359 | (0.12/0.06/0.17) | + |
| H10N5 | A/Zhejiang/CNIC-ZJU01/2023 | 0.1091 | 0.0525 | 0.3267 | (0.12/0.06/0.17) | + |
| H10N7 | A/chicken/Zhejiang/2CP8/2014 | 0.0968 | 0.0566 | 0.3312 | (0.12/0.06/0.17) | + |
| H10N8 | A/chicken/Zhejiang/102615/2016 | 0.0774 | 0.0575 | 0.3238 | (0.12/0.06/0.17) | + |
| H1N1 | A/California/07/2009 | 0.0868 | 0.0546 | 0.1156 | (0.12/0.06/0.17) | – |
| H2N8 | A/duck/Zhejiang/6D10/2013 | 0.0786 | 0.0585 | 0.1042 | (0.12/0.06/0.17) | – |
| H3N2 | A/duck/Zhejiang/4613/2013 | 0.0745 | 0.0502 | 0.0875 | (0.12/0.06/0.17) | – |
| H4N2 | A/chicken/Zhejiang/727145/2014 | 0.0952 | 0.0458 | 0.1094 | (0.12/0.06/0.17) | – |
| H4N6 | A/duck/Zhejiang/409/2013 | 0.1044 | 0.0435 | 0.0813 | (0.12/0.06/0.17) | – |
| H6N1 | A/chicken/Zhejiang/1664/2017 | 0.1091 | 0.0482 | 0.0918 | (0.12/0.06/0.17) | – |
| H9N2 | A/chicken/Zhejiang/61174/2017 | 0.0909 | 0.0579 | 0.0876 | (0.12/0.06/0.17) | – |
| H11N3 | A/duck/Zhejiang/727D2/2013 | 0.085 | 0.0423 | 0.0938 | (0.12/0.06/0.17) | – |
| Marek’s disease virus (MDV) | FC-126 | 0.094814 | 0.049734 | 0.106812 | (0.12/0.06/0.17) | – |
| Avian pox virus (APV) | Quail-Adapted strain | 0.084754 | 0.049258 | 0.101602 | (0.12/0.06/0.17) | – |
| Avian paramyxovirus-4 (APV-4) | ZJ-1 | 0.0957 | 0.0591 | 0.0829 | (0.12/0.06/0.17) | – |
| Infectious bronchitis virus (IBV) | H120 | 0.1085 | 0.0521 | 0.0949 | (0.12/0.06/0.17) | – |
| Infectious bursal disease virus (IBDV) | NF8 | 0.1025 | 0.0553 | 0.1057 | (0.12/0.06/0.17) | – |
| Newcastle disease virus (NDV) | La Sota | 0.1082 | 0.0411 | 0.112 | (0.12/0.06/0.17) | – |
| Infectious laryngotracheitis virus (ILV) | K317 | 0.0848 | 0.0452 | 0.1026 | (0.12/0.06/0.17) | – |
| Severe acute respiratory syndrome coronavirus 2 (SARS-CoV-2) | - | 0.097 | 0.0468 | 0.1165 | (0.12/0.06/0.17) | – |
| Respiratory syncytial virus (RSV) | - | 0.0793 | 0.0419 | 0.0949 | (0.12/0.06/0.17) | – |
| Influenza B virus | - | 0.09545 | 0.050544 | 0.107452 | (0.12/0.06/0.17) | – |
| Rhinovirus | - | 0.096637 | 0.052465 | 0.10103 | (0.12/0.06/0.17) | – |
| Adenovirus | - | 0.08724 | 0.048201 | 0.106629 | (0.12/0.06/0.17) | – |

Note: Cut-off values (0.12 / 0.06 / 0.17) correspond to the detection limits for H5, H7, and H10 subtype AIVs, respectively."+" denotes a positive detection result, and "–" denotes a negative detection result.

**Table S5** Reproducibility of UCNPs-LFA at the LOD level for detection of H5, H7, and H10 subtype AIVs.

|  |  | 2^-4^ HAU | | |  | 2^-3^ HAU | | |  |
| --- | --- | --- | --- | --- | --- | --- | --- | --- | --- |
|  |  | T1/C | T2/C | T3/C |  | T1/C | T2/C | T3/C |  |
| Intra-assay  (n = 10) | Mean | 0.1251 | 0.07256 | 0.1258 |  | 0.1745 | 0.1047 | 0.1739 |  |
|  | SD | 0.0033 | 0.0015 | 0.0049 |  | 0.0028 | 0.0022 | 0.0024 |  |
|  | CV (%) | 2.634 | 2.109 | 3.859 |  | 1.594 | 2.079 | 1.389 |  |
| Inter-assay  (n = 10) | Mean | 0.1243 | 0.07187 | 0.1271 |  | 0.1759 | 0.1027 | 0.1749 |  |
|  | SD | 0.0030 | 0.0013 | 0.0038 |  | 0.0025 | 0.0024 | 0.0018 |  |
|  | CV (%) | 2.389 | 1.797 | 2.984 |  | 1.445 | 2.356 | 1.031 |  |

Note:2^-4^ HAU (2.51×10^2^ TCID_50_/mL for H5 and 1.00×10^2^TCID_50_/mL for H7) and 2^-3^ HAU (5.56×10^2^ TCID_50_/mL for H10)
