# Supplementary figures and images for: Smartphone-assisted upconversion nanoparticle assay for rapid multiplex detection of H5, H7, and H10 avian influenza viruses
Source: Emerg Microbes Infect. 2025 Dec 9;15(1):2602315. doi: 10.1080/22221751.2025.2602315 (PMC12777912; doi:10.1080/22221751.2025.2602315)

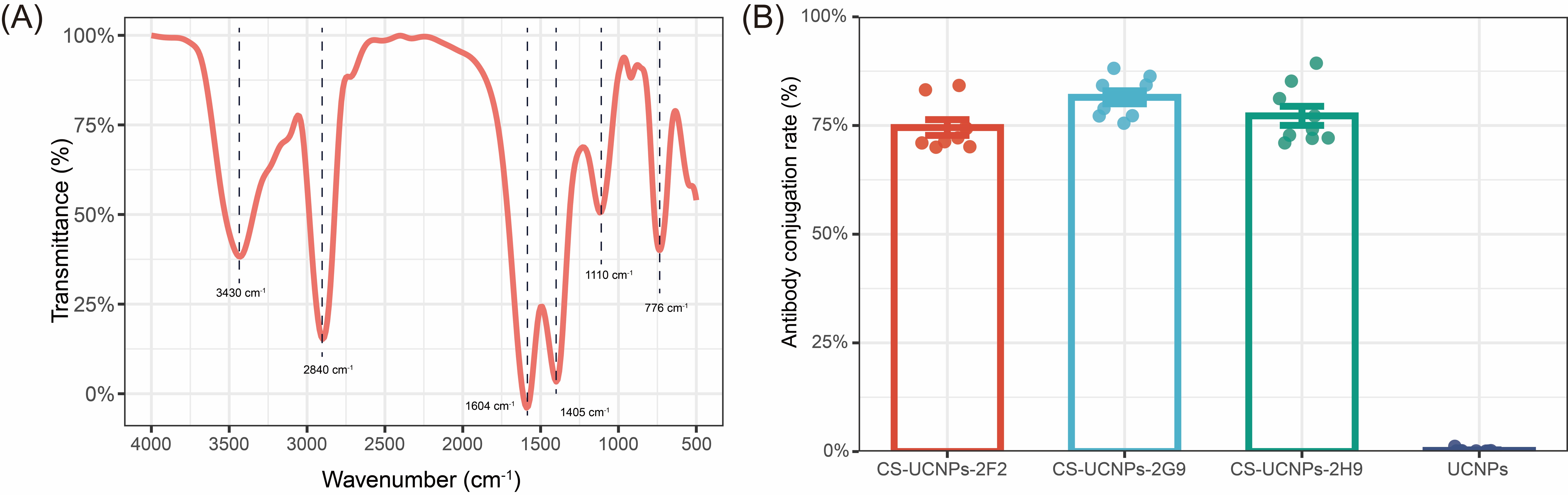

Supplement: Figure S2.jpg [file TEMI_A_2602315_SM9128.jpg]

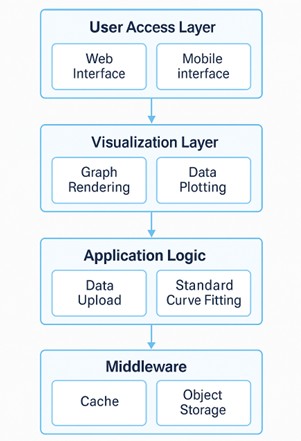

Supplement: Figure S1.jpg [file TEMI_A_2602315_SM9127.jpg]
